# Long noncoding RNA IRL regulates NF-κB-mediated immune responses through suppression of miR-27c-3p-dependent IRAK4 downregulation in teleost fish

**DOI:** 10.1016/j.jbc.2021.100304

**Published:** 2021-01-16

**Authors:** Weiwei Zheng, Qing Chu, Tianjun Xu

**Affiliations:** 1 Laboratory of Fish Molecular Immunology, College of Fisheries and Life Science, Shanghai Ocean University, Shanghai, China; 2 Laboratory of Marine Biology and Biotechnology, Qingdao National Laboratory for Marine Science and Technology, Qingdao, China; 3 Key Laboratory of Exploration and Utilization of Aquatic Genetic Resources (Shanghai Ocean University), Ministry of Education, Shanghai, China; 4 National Pathogen Collection Center for Aquatic Animals, Shanghai Ocean University, Shanghai, China

**Keywords:** long noncoding RNA, microRNA, ceRNA, IRAK4, innate immune

## Abstract

Growing pieces of evidence show that the long noncoding RNAs (lncRNAs) as new regulators participate in the regulation of various physiological and pathological processes. The study of lncRNA in lower invertebrates is still unclear compared with that in mammals. Here, we identified a novel lncRNA, termed IRAK4-related lncRNA (IRL), as a key regulator for innate immunity in teleost fish. We find that miR-27c-3p inhibits IRAK4 expression and thus weakens the NF-κB-mediated signaling pathway. Furthermore, the Gram-negative bacterium *Vibrio anguillarum* and lipopolysaccharide significantly upregulated host lncRNA IRL expression. Results indicate that IRL functions as a competing endogenous RNA for miR-27c-3p to regulate protein abundance of IRAK4; thus, invading microorganisms are eliminated and immune responses are promoted. Our study also demonstrates the regulation mechanism that lncRNA IRL can competitively adsorb miRNA to regulate the miR-27c-3p/IRAK4 axis that is widespread in teleost fish.

Innate immunity is the host's first line of defense against the invasion of pathogenic microorganisms. The pattern recognition receptors (PRRs), as crucial receptor molecule in the innate immune signal transmission, play crucial roles in protecting the host from pathogen invasion. Toll-like receptors (TLRs), RIG-like receptors, and nucleotide oligomerization domain-like receptors are the most important types of PRRs, and they play an irreplaceable role in helping the host identify and monitor various invasive microorganisms (1, 2). TLRs, as one of the most extended receptors, play an irreplaceable role in recognizing and helping to eliminate the invasion of bacteria and viruses (3, 4). Moreover, nearly all TLRs (except TLR3) can activate transcription factors NF-κB and IRF3 in downstream signaling pathways through the interaction of myeloid differentiation factor 88 (MyD88) with IL-1 receptor-associated kinases (IRAKs) (5, 6). Ultimately, these transcription factors activate the host’s antibacterial and inflammatory responses.

IRAK4, as a member of IRAKs, is a crucial serine/threonine kinase involved in the TLR/IL-1R stimulation transduction pathway (7). IRAK4, as a central kinase in the TLR signaling pathway, can bind to MyD88 to form dimers that induce IRAK-1-related phosphorylation; these dimers are recruited into the TLR/IL-1R complex to mediate the NF-κB signaling pathway and induce inflammatory factors and chemokines to fight bacterial invasion (8, 9, 10, 11, 12). The eradication of pathogenic infections requires timely and appropriate immune and inflammatory responses. However, excessive induction of inflammatory cytokines can lead to acute or chronic inflammatory diseases. Recent studies have shown that IRAK4 is associated with some autoimmune diseases. Umar *et al*. (13) found that IRAK4 inhibitor therapy attenuated rheumatoid arthritis disease activity by blocking TLR7-induced M1-MΦ or fibroblasts activation, as well as monokine-modulated Th1/Th17 cell polarization. Recent studies have also found that suppressed IRAK4 can inhibit plasmacytoid dendritic cells and natural killer cells from producing cytokines to treat systemic lupus erythematosus, which is an autoimmune disease that activates the circulating immune complexes and type I interferon and then produces proinflammatory cytokines and chemokines; this phenomenon to autoimmune reactions and organ inflammations (14). Studies show that IRAK4-deficient children are susceptible to life-threatening pyogenic infections; therefore, IRAK4 is essential for the innate immune system (7, 15). Similar to the situation in mammals, our previous study suggested that IRAK4 is a key mediator involved in the host's innate immune response of the host in fish (16). IRAK4 plays a key role in inflammatory cytokine induction and innate immune response. Thus, further exploration of the regulatory mechanism of IRAK4-mediated signal transduction is urgently needed.

Noncoding RNA (ncRNA) is defined as a collection of RNAs that do not have the potential to encode proteins (17). Long ncRNAs (lncRNAs), microRNAs (miRNAs), and circular RNAs are the three types of noncoding RNAs that have been studied comprehensively in mammals (18, 19, 20). LncRNAs were first discovered in large-scale sequencing of mouse full-length enriched cDNA library (21). Considering their unexpected abundance, lncRNAs were originally thought to be pseudotranscriptional noise caused by low RNA polymerase fidelity considering their unexpected abundance (22). However, increasing evidence has shown that lncRNAs can play a critical regulator involved in higher-order chromosome dynamics, telomere biology, and subcellular structure organization (23). Recent studies also show that lncRNAs regulate targets via different mechanistic models. Some lncRNAs directly interact with heteronuclear ribonucleo proteins or chromatin modification complexes, whereas some lncRNAs function as competing endogenous RNAs (ceRNAs) and cross talk with mRNAs by competing for shared miRNAs (24, 25, 26). ceRNA regulatory networks have been widely investigated in mammals, such as humans and mice, to date. Although considerable literature has proposed that an increasing number of lncRNAs have been annotated in vertebrates, the question of whether the ceRNA regulatory networks are widespread in all vertebrates is of particular interest for researchers.

miRNAs are a class of highly conserved ncRNAs with a length of only 21 to 24 nucleotides (27). The in-depth study of miRNA in recent decades has shown that miRNA has an irreplaceable important role in organisms, from lower vertebrates to mammals. Studies have shown that miRNA can participate in various physiological and pathological development processes, such as regulating cell proliferation, growth, apoptosis, and differentiation (28). miRNAs repress gene expression through binding to the 3’-untranslated region (UTR) of the target mRNAs by inhibiting mRNA translation or promoting mRNA degradation. In addition, recent studies show that miRNAs have many other regulatory mechanisms, such as the more popular ceRNA network mechanism. In this mechanism, there are many miRNA response elements on lncRNA molecules, so it can be used as a molecular sponge to competitively bind miRNA to regulate mRNA (29). This ceRNA mechanism was first discovered in *Arabidopsis* by Franco-Zorrilla *et al* (30). They found that lncRNA IPS1 can regulate the expression of PHO2 gene by competitively sponging miR-399. Subsequently, increasing evidence has shown that the ceRNA mechanism of lncRNA-miRNA-mRNA exists both in plants and mammals (31). However, the lncRNA-miRNA regulatory mechanism has been limited to be found in plants and mammals, and little is known whether the mechanism exists in lower vertebrates, such as fish.

In this study, we first identified a ceRNA regulatory loop involved in IRAK4-mediated NF-κB signaling in teleost fish. Our results suggested that the miR-27c-3p can regulate IRAK4 expression and suppress the IRAK4-mediated immune response. We demonstrated that a lncRNA, named IRL, can act as a ceRNA for miR-27c-3p to facilitate IRAK4 expression; as a result, immune responses are modulated. Our research not only contributes to the understanding of the ceRNA network mechanism in teleost fish but also provides a reference for the importance of lncRNA in the innate immune response in lower vertebrates.

## Experimental procedures

### Sample and challenge

Miiuy croaker, *Miichthys miiuy* (∼50 g) was obtained from Zhoushan Fisheries Research Institute, Zhejiang Province, China. Fish were acclimated in aerated seawater tanks at 25 °C for 6 weeks before experiments. The bacterial challenge was performed as described (32). Briefly, fish was challenged with 0.2 ml of *Vibrio anguillarum* (1.5 × 10^8^ cfu/ml) or 0.2 ml suspension of lipopolysaccharide (LPS) (1 mg/ml) intraperitoneally. As comparison, 0.2 ml of physiological saline was used to challenge the individuals. Afterward, fish were respectively sacrificed at different time points and the spleen samples were collected for RNA extraction. All animal experimental procedures were performed in accordance with the National Institutes of Health’s Guide for the Care and Use of Laboratory Animals, and the experimental protocols were approved by the Research Ethics Committee of Shanghai Ocean University (No. SHOU-DW-2018-047).

### Cell culture and treatment

*M. miiuy* intestine cells (MICs) and *M. miiuy* kidney cells were cultured in L-15 medium (HyClone) supplemented with 15% fetal bovine serum (Gibco), 100 U/ml penicillin, and 100 μg/ml streptomycin at 26 °C. *Epithelioma papulosum cyprinid* (EPC) cells were maintained in medium 199 (Invitrogen) supplemented with 10% fetal bovine serum, 100 U/ml penicillin, and 100 mg/ml streptomycin at 26 °C in 5% CO_2_. For stimulation experiments, MICs were challenged with an LPS concentration of 10 μg/ml and harvested at different times for RNA extraction (33).

### Plasmid construction and cell transfection

To construct IRL and IRAK4 3’UTR luciferase genes, the sequences of IRL and IRAK4 3’UTR in *M. miiuy*, *Nibea diacanthus*, and *Larimichthys crocea* were cloned into pmirGLO luciferase reporter vector, respectively. The mutated forms with point mutations in the miR-27c-3p-binding site were synthesized using Mut Express II Fast Mutagenesis Kit V2 with specific primers (Table S1). Meanwhile, the sequences of IRL were inserted into the mVenus-C1 vector (Invitrogen), which included the sequence of enhanced GFP. Also, IRL and IRAK4 expression plasmids were constructed by cloning the IRL and IRAK4 sequence region of *M. miiuy*, *N. diacanthus*, and *L. crocea* into the pcDNA3.1 vector, respectively. To build pcDNA3.1-MS2, the MS2-12X fragment was inserted into the *BamH* I and *EcoR* V restriction sites of pcDNA3.1 vector, and then the IRL was amplified and cloned into pcDNA3.1-MS2. The mutated forms with point mutations in the miR-27c-3p-binding site were synthesized using Mut Express II Fast Mutagenesis Kit V2 with specific primers (Table S1). An miR-27c-3p sensor was created by inserting two consecutive miR-27c-3p complementary sequences into the psiCHECK vector (Promega). The correct construction of the plasmids was verified by Sanger sequencing and extracted through EndoFree Plasmid DNA Miniprep Kit (Tiangen). Transfections were performed using the Lipofectamine RNAiMAX and Lipofectamine 3000 (Invitrogen) according to the manufacturer’s instructions, respectively.

### RNA oligoribonucleotides

The miR-27c-3p mimics are synthetic double-stranded RNAs (dsRNAs) with stimulating naturally occurring mature miRNAs. The miR-27c-3p mimics sequences were 5’-UUCACAGUGGUUAAGUUCUGC-3’ (sense) and 5’-AGAACUUAACCACUGUGAAUU-3’ (antisense). The negative control RNA sequences were 5’-UUCUCCGAACGUGUCACGUTT-3’ (sense) and 5’-ACGUGACACGUUCGGAGAATT-3’ (antisense). miRNA inhibitors are synthetic single-stranded RNAs (ssRNAs) that sequester intracellular miRNAs and block their activity in the RNA interfering pathway. The miR-27c-3p inhibitor sequence was 5’-GCAGAACUUAACCACUGUGAA-3’. The negative control inhibitor sequence was 5’-CAGUACUUUUGUGUAGUACAA-3’. The RNA interference for IRL are as follows: the si-IRL-1 sequence was 5’-ACGUCAUCACUGUCACAUCTT-3’; the si-IRL-2 sequence was 5’- CGAUCAGGUUAAUCUUCUATT-3; the negative control RNA sequences were 5’-UUCUCCGAACGUGUCACGUTT-3’ (sense) and 5’-ACGUGACACGUUCGGAGAATT-3’ (antisense). The RNA interference for IRAK4 is as follows: the si-IRAK4 sequences were 5’-GCAUCAUGUGAGGAGGUUUTT-3’ (sense) and 5’-AAACCUCCUCACAUGAUGCTT-3’ (antisense). The scrambled control RNA sequences were 5’-UUCUCCGAACGUGUCACGUTT-3’ (sense) and 5’-ACGUGACACGUUCGGAGAATT-3’ (antisense).

### RNA extraction and quantitative real-time PCR

Both nuclear RNA and cytoplasmic RNA were extracted from MICs using the Cytoplasmic & Nuclear RNA Purification Kit (Norgen Biotek) according to the manufacturer’s instructions. Total RNA was isolated using TRIzol Reagent (Invitrogen), and cDNA was obtained by reverse transcriptional RNA using FastQuant RT Kit (Tiangen), which includes DNase treatment of RNA to eliminate genomic contamination. The nuclear and cytosolic fractions were separated using the Cytoplasmic & Nuclear RNA Purification Kit (Norgen Biotek) according to the manufacturer’s instructions. Gene expression was measured by using SYBR Premix Ex TaqTM (Takara). The small RNA was extracted by using miRcute miRNA Isolation Kit (Tiangen), and miRcute miRNA FirstStrand cDNA Synthesis Kit (Tiangen) was applied to reverse transcription of miRNAs. The expression analysis of miR-27c-3p was executed by using the miRcute miRNA qPCR Detection Kit (Tiangen). Quantitative real-time PCR was performed in an Applied Biosystems QuantStudio 3 (Thermo Fisher Scientific). GAPDH and 5.8S rRNA were used as negative controls to detect mRNA, lncRNA, and miRNA expression, respectively (34). Primer sequences are displayed in Table S1.

### Dual-luciferase reporter assays

The IRL or IRAK4-3’UTR wildtype and the mutant devoid of the miR-27c-3p-binding site were cotransfected with miR-27c-3p mimics into EPC cells. At 24 h post transfection, reporter luciferase activities were measured using the Dual-Luciferase reporter assay system (Promega). To determine the functional regulation of IRL or IRAK4, MICs were cotransfected with IRAK4 expression plasmid or IRL expression plasmid, together with NF-κB, IL-8, and IL-1β luciferase reporter gene plasmids (34), phRL-TK Renilla luciferase plasmid, either miR-27c-3p mimics or negative controls. At 48 h post transfection, the cells were lysed for reporter activity using the Dual-Luciferase reporter assay system (Promega). The miR-27c-3p sensor was cotransfected with miR-27c-3p mimics or IRL expression plasmid into MICs. At 48 h post transfection, the cells were lysed for reporter activity. All the luciferase activity values were achieved against the Renilla luciferase control. The transfection of each construct was performed in triplicate in each assay. Ratios of Renilla luciferase readings to firefly luciferase readings were taken for each experiment, and triplicates were averaged.

### Western blotting

Cells were lysed and collected in 1× SDS-PAGE loading buffer. The protein content in the collected cell lysates was measured with the bicinchoninic acid protein detection kit (Vazyme), then subjected to SDS-PAGE (10%) gel and transferred to PVDF (Millipore) membranes by semidry blotting (Bio-Rad Trans Blot Turbo System). The membrane is sealed with 5% bovine serum albumin. Protein was detected with different antibodies. The antibody against IRAK4 was diluted at 1:1000 (Genscript); anti-FLAG, anti-GFP, and anti-Tubulin monoclonal antibodies were diluted at 1: 2000 (Beyotime); and horseradish peroxidase–conjugated anti-rabbit IgG or anti-mouse IgG (Abbkine) were diluted at 1: 5000. The results were representative of three independent experiments. The immunoreactive proteins were detected by using WesternBright ECL (Advansta). The digital imaging was performed with a cold CCD camera.

### RNA pull-down assay

IRL and IRL-mut with miR-27c-3p-binding sites mutated were transcribed *in vitro*. The two transcripts were biotin labeled with the T7 RNA polymerase and Biotin RNA Labeling Mix (Roche), treated with RNase-free DNase I, and purified with an RNeasy Mini Kit (Qiagen). The whole-cell lysates from MICs (∼1.0 × 10^7^) were incubated with purified biotinylated transcripts for 1 h at 25 °C. The complexes were isolated by streptavidin agarose beads (Invitrogen). RNA was extracted from the remaining beads, and qPCR was used to evaluate the expression levels of miRNAs. The specific protocol of pull-down assay was as described (35).

### RNA immunoprecipitation assay

To explore whether IRL is involved in the ceRNA regulation process, MICs (∼2.0 × 10^7^) were cotransfection with pcDNA3.1-FLAG, pcDNA3.1-AGO2-FLAG, or miR-27c-3p for RNA immunoprecipitation (RIP) assays. To prove that IRL has the ability to directly bind to miR-217, MICs (∼2.0 × 10^7^) were cotransfected with pcDNA3.1-MS2, pcDNA3.1-MS2-IRL, pcDNA3.1-MS2-IRL-mut, pcDNA3.1-MS2-IRAK4-3’UTR, or pMS2-GFP for RIP assays. In short, MICs were harvested after 48 h transfection and RIP assays were carried out with Magna RIP RNA-Binding Protein Immunoprecipitation Kit (Millipore) and anti-GFP antibody (Abcam) following the manufacturer’s protocol. RNA was extracted from the beads, and the expression level of miRNA was evaluated by qRT-PCR.

### Cell viability and proliferation

Cell viability was measured 48 h after transfection in LPS-treated MIC with Celltiter-Glo Luminescent Cell Viability assays (Promega) according to the manufacturer’s instructions. At the same time, cell proliferation was measured with BeyoClick EdU cell Proliferation Kit following the manufacturer’s instructions (Beyotime). All the experiments were performed in triplicate.

### Flow cytometric analysis of apoptosis

The apoptotic assay was conducted using an Annexin V-FITC Apoptosis Detection Kit (Beyotime) and analyzed with flow cytometry (Beckman). The ratio of early and late apoptotic cells was detected to calculate the apoptotic rate (36).

### Statistical analysis

Data are expressed as the mean ± SD from at least three independent triplicated experiments. Student’s *t* test was used to evaluate the data. The relative gene expression data were acquired using the 2 ^ΔΔCT^ method, and comparisons between groups were analyzed by one-way analysis of variance (ANOVA) followed by Duncan’s multiple comparison tests (37). The value of *p* < 0.05 was considered significant.

## Result

### Characterization of lncRNA IRL

**Figure 1:**
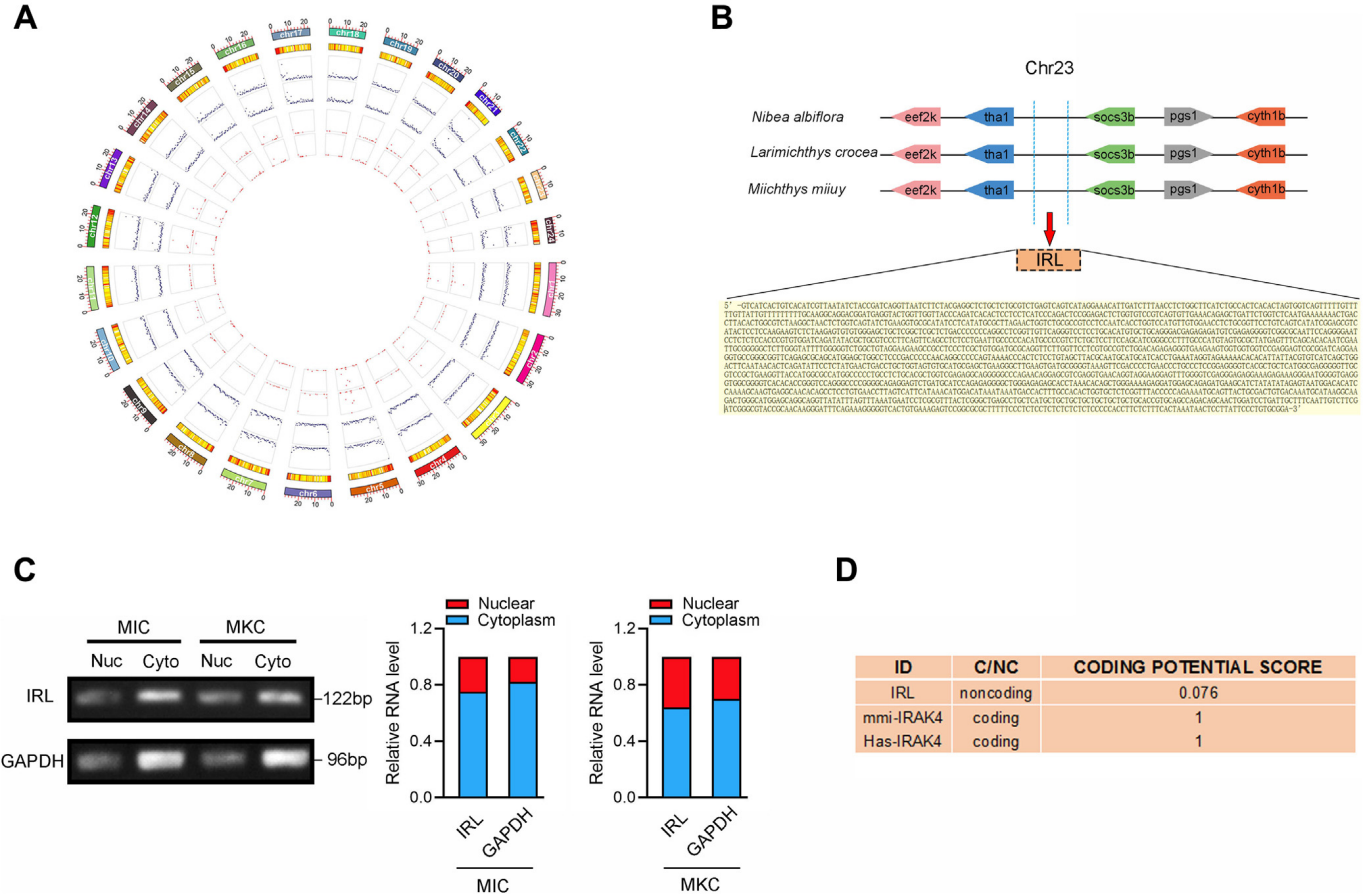
**Characterization of lncRNA IRL.***A*, circos plot displayed the distribution and expression of lncRNAs on miiuy croaker chromosomes. The outermost layer was a chromosome map of the miiuy croaker genome. The outermost two inner rings from outside to inside correspond to the distribution and expression of low-expression lncRNAs treated with *V. anguillarum* and untreated spleen tissues, respectively. And the innermost two rings from the outside to the inside correspond to the distribution and expression of highly expressed lncRNAs in *V. anguillarum*-treated and untreated spleen tissues, respectively. *B*, schematic of the IRL locus. IRL is located on *M. miiuy*, *N. diacanthus*, and *L. crocea* chromosome 23. *C*, the overexpressed IRL was mainly located in the cytoplasm. *D*, IRL was predicted to be a noncoding RNA. The RNA sequences of IRL was put into the Coding Potential Calculator program, which was predicted to be noncoding RNAs. *mmi*-IRAK4, *M. miiuy* IRAK4 gene; *hsa*-IRAK4, *Homo sapiens* IRAK4 gene; IRL, IRAK4-related lncRNA.

We treated miiuy croaker with *V. anguillarum* to identify whether lncRNAs are potentially involved in the regulation of bacterial infection, and then, the expression of lncRNAs in the spleen tissues of the treated group and the untreated group was analyzed by RNA-Seq data. The circus plot displayed the distribution and expression of detected and significantly expressed lncRNAs on miiuy croaker chromosomes (Fig. 1*A*). IRL that is one of the significantly different upregulated lncRNAs has attracted our attention. The single-molecule full-length transcript sequencing was used to characterize the complete sequence of IR. The results demonstrate that the length of IRL is 1891 base pairs (bp) and IRL locates on miiuy croaker, *N. diacanthus*, and *L. crocea* chromosome 23, between gene *tha1* and gene *socs3b*, and consists of only one exon (Fig. 1*B*). Then, we detected the subcellular location of IRL and found that IRL is mainly expressed in the cytoplasm (Fig. 1*C*). Consistent with IRL being an ncRNA, there was no putative protein conserved in all species, and the CPC (coding potential calculator) computational algorithm predicts that IRL has a very low coding potential (Fig. 1*D*).

### IRL enhances the host antibacterial responses

**Figure 2:**
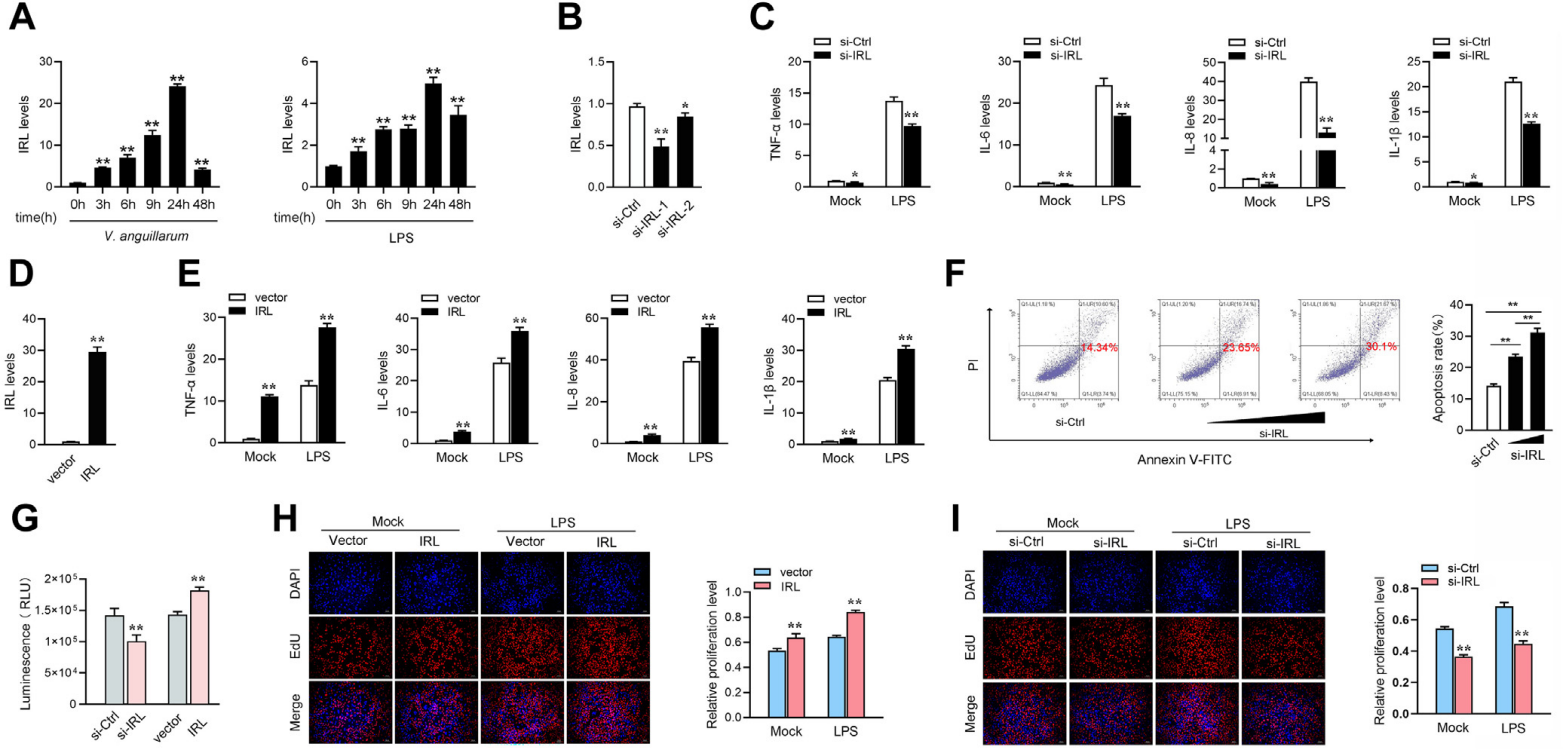
**IRL enhances host antibacterial responses.***A*, *V. anguillarum* and LPS induces an increase of IRL expression. The expression levels of IRL in spleen was measured by qPCR at the indicated time after *V. anguillarum* infection or LPS treatment. *B*, the effect of si-IRL on endogenous IRL expression. MICs were transfected with IRL-specific siRNAs (si-IRL-1 and si-IRL-2) or si-Ctrl (*B*) for 48 h, then IRL expression was determined by qPCR. *C*, MICs were transfected with si-Ctrl or si-IRL-1. At 48 h post transfection, MICs were treated with LPS for 6 h. The expression of TNF-α, IL-6, IL-8, and IL-1β was analyzed by qPCR. *D*, the effect of IRL expression plasmid on endogenous IRL expression. MICs were transfected with pcDNA3.1 vector or IRL expression plasmid for 48 h, then IRL expression was determined by qPCR. *E*, MICs were transfected with pcDNA3.1 vector or IRL expression plasmid for 48 h. The cells were treated with LPS for 6 h. The expression of TNF-α, IL-6, IL-8, and IL-1β were analyzed by qPCR. *F*, the effect of IRL knockdown on cell apoptosis was analyzed by flow cytometric cell apoptosis assays. *G*, effect of si-IRL and IRL-overexpressed plasmid on cell proliferation and viability. MICs were transfected with si-NC or si-IRL and vector or IRL-overexpressed plasmid for 48 h. Cell viability assay was performed. *H* and *I*, cell proliferation was assessed by EdU assays in MICs transfected with pcDNA3.1 vector or IRL expression plasmid (*G*) and si-IRL or si-NC (*H*) effect of IRL on cell viability after LPS stimulation. MICs were transfected with si-Ctrl, si-IRL, pcDNA3.1 vector, or IRL for 48 h, then treated with LPS for 6 h. Cell proliferation assay was performed. All data represented the mean ± SD from three independent triplicated experiments. ∗*p* < 0.05; ∗∗*p* < 0.01. IRL, IRAK4-related lncRNA; LPS, lipopolysaccharide; MIC, *M. miiuy* intestine cell.

We first detected the IRL expression in the spleen under different *V. anguillarum* or LPS stimulation times to verify the results of RNA-Seq. As shown in Figure 2*A*, we found that the expression of IRL is significantly upregulated after *V. anguillarum* or LPS stimulation. Two small interfering RNAs were designed against IRL (si-IRL-1 and si-IRL-2) and the overexpression plasmid of IRL was constructed to explore the biological functions of IRL. As expected, both siRNAs can inhibit the expression of IRL and si-IRL-1 can induce higher inhibitory efficiency (Fig. 2*B*). The IRL overexpression plasmid could significantly increase IRL expression levels (Fig. 2*D*). Inflammatory cytokines play an important role in the antibacterial immune response; we thus focused on investigating the function of IRL in regulating the expression of inflammatory cytokines. As shown in Figure 2*C*, knockdown of IRL can significantly inhibit the expression levels of TNF-α, IL-6, IL-8, and IL-1β upon LPS stimulation. On the contrary, overexpression of IRL increases these gene expression levels in MICs under LPS treatment (Fig. 2*E*). Furthermore, cell apoptosis analysis showed that IRL knockdown increased the proportion of apoptotic cells (Fig. 2*F*). We conducted EdU assays and ATP activity assay to examine cell proliferation and cell viability for further exploring the role of IRL in innate immunity. Furthermore, we tested the effects of si-IRL and IRL-overexpressed plasmid on MIC viability. The results showed that knockdown of IRL decreases the cell viability, whereas it greatly increased when IRL-overexpressed plasmid is transfected (Fig. 2*G*). As shown in Figure 2, *H* and *I*, the results showed that knockdown of IRL considerably decreased the percentage of EdU-positive cells and cell viability, whereas it greatly increased when IRL is overexpressed. These results suggested that IRL can promote the proliferation and viability in miiuy croaker cell lines. Of interest, our results are in perfect agreement with some reports that low concentrations of LPS can stimulate cell proliferation (38). In summary, these data suggest that IRL serves as a positive modulator in regulating inflammatory responses and cell proliferation and cell viability. The changes in cell proliferation and cell apoptosis are a result of the regulation effect of IRL on innate immune responses. In other words, IRL can positively regulate the antibacterial responses and upregulate the expression of inflammatory cytokines, reducing the bacterial attack of cells and promoting cell proliferation, and reducing cell apoptosis.

### IRL can regulate miR-27c-3p expression and activity

**Figure 3:**
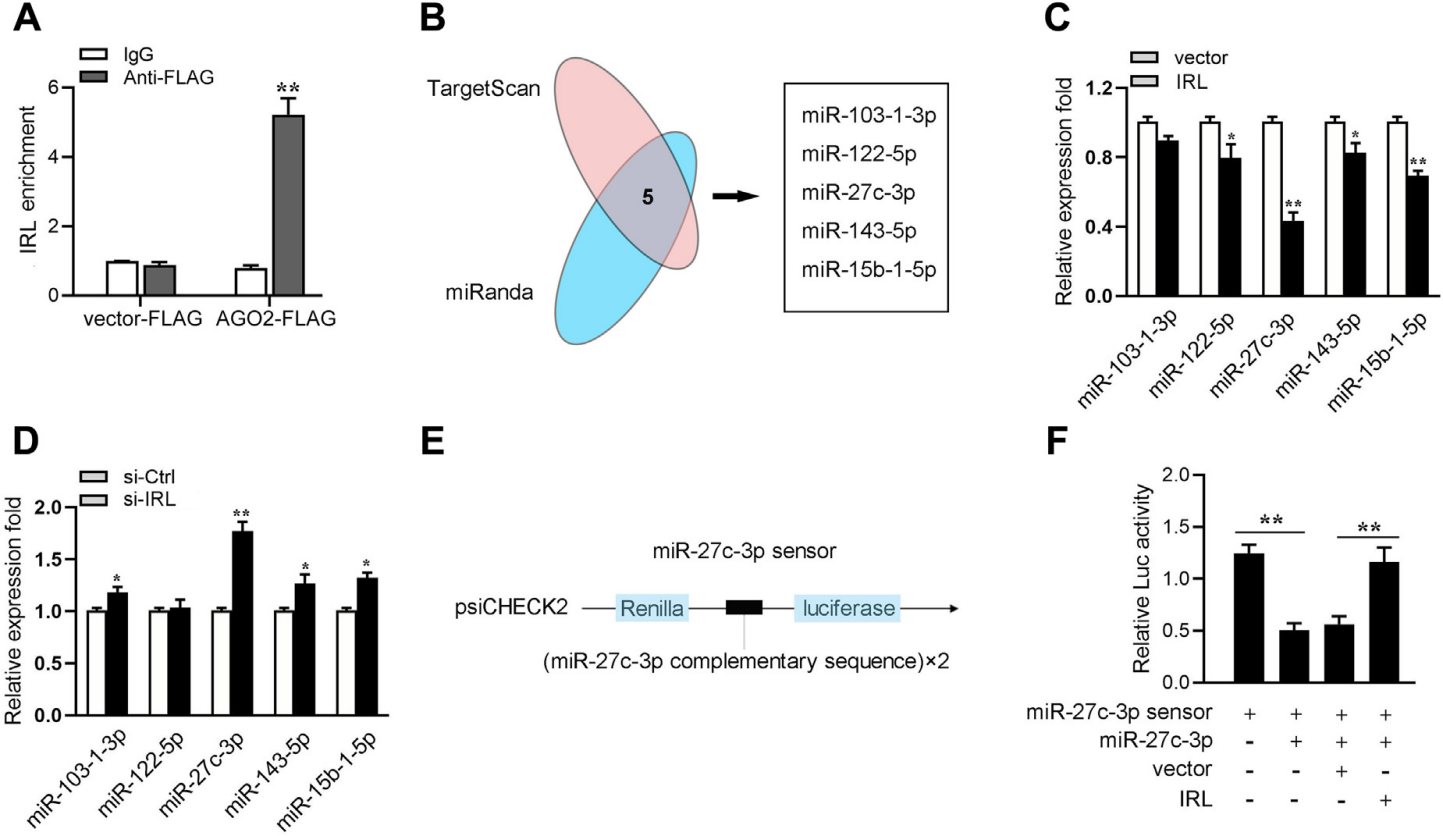
**IRL can regulate miR-217-3p expression and activity.***A*, the Ago-RIP assay for the amount of IRL in MICs transfected Ago2-FLAG or pcDNA3.1-FLAG. *B*, a schematic illustration showing overlapping of the target miRNAs of IRL predicted by TargetScan and miRanda. *C* and *D*, relative expression of candidate miRNAs in MICs transfected with IRL expression plasmid (*C*) and si-IRL (*D*), respectively. *E*, miR-27c-3p sensor construct. The miR-27c-3p sensor was constructed by inserting two copies of perfectly matched miR-27c-3p fragments into psiCHECK-2 vector. *F*, IRL reduces miR-27c-3p activity. MICs were transfected with mimics, control vector, or IRL overexpression plasmid, together with miR-27c-3p sensor. The luciferase activity was analyzed and normalized to Renilla luciferase activity. All data represented the mean ± SD from three independent triplicated experiments. ∗*p* < 0.05; ∗∗*p* < 0.01. IRL, IRAK4-related lncRNA; MIC, *M. miiuy* intestine cell.

Many cytoplasmic lncRNAs have been reported to act as ceRNAs by competitively binding miRNAs (33, 35). We determine from the subcellular localization experiments that IRL is mainly distributed in the cytoplasm. Therefore, to further confirm whether IRL acts as a ceRNA, we next constructed the RIP assay with Ago2-FLAG in MICs. As shown in Figure 3*A*, IRL is significantly enriched in Ago2-containing microribonucleoprotein complexes, which suggests that the miRNAs can directly bind to IRL. Then, we used TargetScan and miRanda software to predict miRNAs that can potentially target IRL, and the five microRNAs (including miR-27c-3p, miR-103-5p, miR-143-5p, miR-221-5p, and miR-92-5p) were predicted to have a high probability of combining to IRL (Fig. 3*B*). Then, we compared the expression levels of these candidate miRNAs in MICs transfected with si-IRL or negative controls and IRL overexpression plasmid or control vector. Among the five candidate miRNAs, miR-27c-3p is significantly enhanced in response to IRL inhibition and is markedly impaired in response to IRL overexpression (Fig. 3, *C* and *D*). We constructed the miR-27c-3p sensor to test whether IRL can affect miR-27c-3p activity. The miR-27c-3p sensor was constructed by inserting two copies of perfectly matched miR-27c-3p fragments into psiCHECK vector (Fig. 3*E*). Then, we transfected it with the miR-27c-3p sensor into cells, along with miR-27c-3p, control vector, or IRL overexpression plasmid; notably, IRL specifically sponges miR-27c-3p, which prevents it from inhibiting luciferase activity (Fig. 3*F*). Overall, these data suggest that IRL can regulate miR-27c-3p expression and activity, and IRL may function as an miRNA sponge to adsorb and consume the miR-27c-3p.

### IRL can bind miR-27c-3p

**Figure 4:**
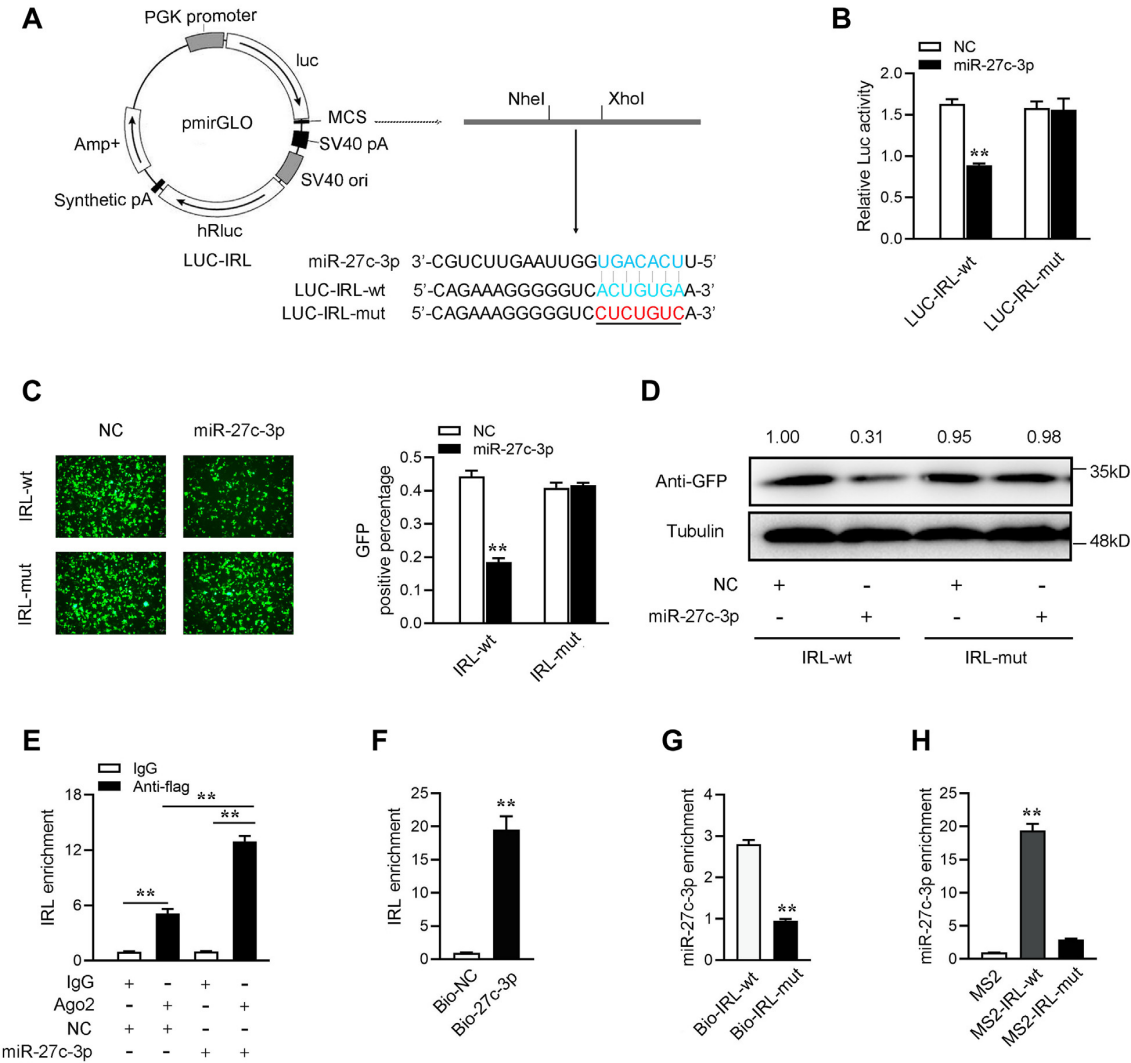
**IRL is able to bind miR-27c-3p.***A*, the IRL sequence contains one site complementary to miR-27c-3p. miR-27c-3p-binding sites in IRL wildtype form (Luc-IRL-wt) and the mutated form (Luc-IRL-mut) were shown. *B*, epithelioma papulosum cyprinid cells were transfected with NC or miR-27c-3p, together with Luc-IRL-wt or Luc-IRL-mut. The luciferase activity was analyzed and normalized to Renilla luciferase activity. *C* and *D*, miR-27c-3p could downregulate GFP expression. Epithelioma papulosum cyprinid cells were cotransfected with the wildtype of mVenus-IRL or the mutated type, together with NC or miR-27c-3p. At 48 h post transfection, the fluorescence intensity (*C*) and the GFP expression (*D*) were evaluated by enzyme-labeled instrument and Western blotting, respectively. The scale bar represents 20 μm; original magnification ×10. *E*, the Ago-RIP assay for the amount of IRL in MICs transfected with Ago2-FLAG and miR-27c-3p or pcDNA3.1-FLAG and NC. *F* and *G*, RNA pull-down assay was executed in MICs, followed by qPCR to detect the enrichment of IRL and miR-27c-3p. *H*, the qPCR results of the MS2-RIP method used to identify the binding between IRL and miR-27c-3p in MICs. miRNA real-time qPCR was performed after RNA immunoprecipitation process (right panel). All data represented the mean ± SD from three independent triplicated experiments. ∗*p* < 0.05; ∗∗*p* < 0.01. IRL, IRAK4-related lncRNA; MIC, *M. miiuy* intestine cell.

We analyzed the sequence of IRL to investigate whether IRL can directly interact with miR-27c-3p and find a binding site for miR-27c-3p. Then, we constructed the IRL luciferase plasmid and the mutant plasmids with the mutated miR-27c-3p-binding site (Fig. 4*A*). The luciferase assay showed that miR-27c-3p mimics can significantly inhibit the wildtype IRL luciferase plasmid activity but has no effect on the mutated form (Fig. 4*B*). In addition, we inserted the wildtype or the mutant type fragment of IRL into the mVenus-C1 vector and cotransfected with miR-27c-3p to confirm whether miR-27c-3p can inhibit the levels of GFP. As shown in Figure 4*C*, miR-27c-3p can significantly inhibit the levels of GFP but cannot inhibit the level of the mutant type of IRL-GFP. To extend the findings, the Western blotting assay has been conducted to examine the expression level of GFP protein (Fig. 4*D*). These results indicate that IRL may interact with miR-27c-3p through the predicted miR-27c-3p-binding site.

We determined through the previous RIP experiment that IRL can directly bind to miRNAs (Fig. 3*A*). We thus further tested the ability of IRL to bind to miR-27c-3p. To this end, the Ago2 RIP assays were performed in MICs by cotransfecting Ago2-FLAG and miR-27c-3p. The results from qRT-PCR analysis suggested that IRL and miR-27c-3p are efficiently pulled down by Ago2-FLAG (Fig. 4*E*). For further confirming the direct interaction between IRL and miR-27c-3p, we performed biotin-avidin pull-down experiments to examine whether miR-27c-3p or IRL can be pulled down in MICs that are transfected with biotinylated miR-27c-3p or biotinylated IRL and can then be harvested for pull-down assay. The result showed that IRL could be pulled down by biotinylated wildtype miR-27c-3p (Fig. 4*F*). The miR-27c-3p can be pulled down by biotinylated wildtype IRL (Fig. 4*G*). Then we constructed plasmids that recognize lncRNAs by the MS2 protein. We inserted the MS2-12X fragment into the pcDNA3.1, pcDNA3.1-IRL, and the mutated type of IRL plasmids (pcDNA3.1-IRL-mut). We constructed a fusion expression plasmid of GFP and MS2 to produce MS2-GFP fusion protein (pcDNA3.1-MS2-GFP), and the protein can bind to MS2-12X and also GFP antibody. Therefore, if miRNAs can bind to IRL, then miR-27c-3p can be pulled down by the GFP-MS2-12X complex. Analysis of qPCR results showed that the miR-27c-3p enrichment of IRL is significantly higher than that of mutant IRL and empty plasmid (Fig. 4*H*). In summary, these results indicated that IRL can directly bind to miR-27c-3p and IRL may act as a sponge of miR-27c-3p.

### miR-27c-3p suppresses innate immunity by targeting IRAK4

We first examined the miR-27c-3p expression profiles in miiuy croaker to identify whether miR-27c-3p is involved in the immune response induced by *V. anguillarum*. As shown in Figure S1, we found the expression of miR-27c-3p in spleen tissues is significantly increased after *V. anguillarum* stimulation. Then, we first searched for predicted miR-27c-3p targets using bioinformatics tools (Table S2) to identify the molecular target of miR-27c-3p. Among the possible targets of miR-27c-3p, we focused on IRAK4, which has been repeatedly demonstrated to be a critical player involved in TLR-dependent immune response. We found that IRAK4 can promote cell proliferation but knocking down IRAK4 promotes apoptosis (Fig. S2).

**Figure 5:**
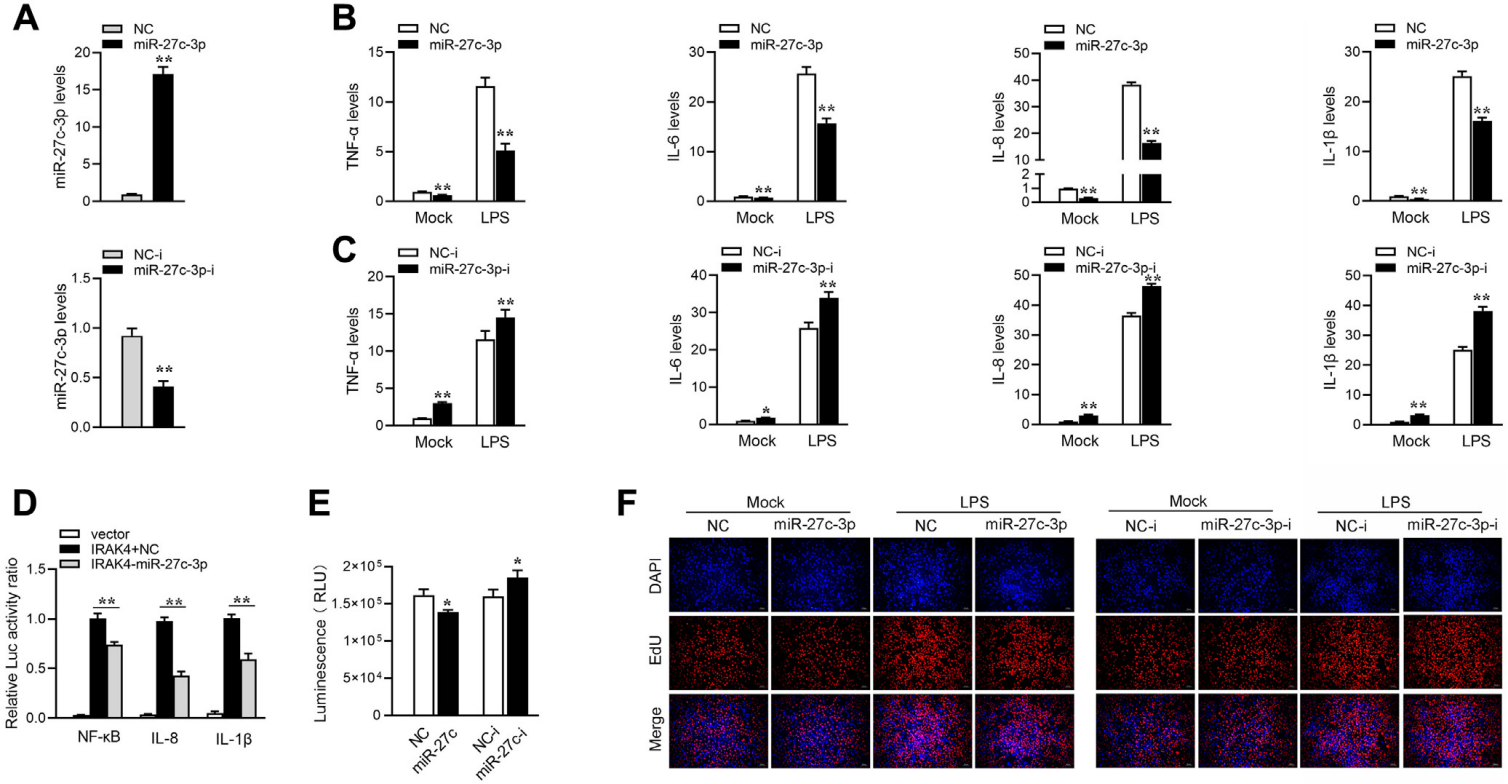
**miR-27c-3p suppresses innate immunity by targeting IRAK4.***A*, the effect of miR-27c-3p mimics and inhibitors on endogenous miR-27c-3p expression. MICs were transfected with NC or miR-27c-3p (*top panel*) and NC-i or miR-27c-3p-i (*bottom panel*) for 48 h, then miR-27c-3p expression was determined by qPCR. *B* and *C*, overexpression of miR-27c-3p attenuates the expression of inflammatory genes. MICs were transfected with NC, miR-27c-3p, NC-i, or miR-27c-3p-i. At 48 h post transfection, the cells were treated with lipopolysaccharide for 6 h. The expression levels of TNF-α, IL-6, IL-8, and IL-1β were analyzed by qPCR. *D*, miR-27c-3p could suppress NF-κB, IL-8, and IL-1βsignaling. MICs were transfected with NC or miR-27c-3p, together with IRAK4 expression plasmid, phRL-TK Renilla luciferase plasmid, and luciferase reporter genes. The luciferase activity was measured and normalized to Renilla luciferase activity. *E* and *F*, effect of miR-27c-3pand miR-27c-3p-i on cell proliferation and viability. MICs were transfected with NC or miR-27c-3p and NC-i or miR-27c-3p-i for 48 h, then treated with lipopolysaccharide. Cell proliferation assay was performed. The scale bar represents 20 μm; original magnification ×40. All data represented the mean ± SD from three independent triplicated experiments. ∗*p* < 0.05; ∗∗*p* < 0.01. MIC, *M. miiuy* intestine cell.

We measured the effects of synthetic miR-27c-3p mimics and miR-27c-3p inhibitors on the expression of miR-27c-3p. As expected, miR-27c-3p mimics enhanced miR-27c-3p expression sharply, whereas miR-27c-3p inhibitors decreased miR-27c-3p expression (Fig. 5*A*). Next, we explored the role of miR-27c-3p against the immune response, and miR-27c-3p and miR-27c-3p inhibitors were transfected into MICs and stimulated by LPS, respectively. The results showed that certain inflammatory cytokines, including TNF-α, IL-6, IL-8, and IL-1β are significantly decreased by the introduction of miR-27c-3p mimics upon LPS stimulation. On the contrary, the inhibition of endogenous miR-27c-3p significantly elevated these gene expressions compared with the transfection of control inhibitors (Fig. 5, *B* and *C*). IRAK4-3’UTR plasmid, miR-27c-3p, and reporter genes were cotransfected into EPC cells to verify that IRAK4 can be regulated by miR-27c-3p. The results showed that miR-27c-3p could inhibit the luciferase activity of inflammatory cytokines by inhibiting IRAK4 (Fig. 5*D*). We tested the effects of miR-27c-3p and miR-27c-3p-i on MIC proliferation and viability. The results showed that miR-27c-3p considerably decreases the percentages of EdU-positive cells and cell viability, whereas they greatly increased when miR-27c-3p-i is transfected (Fig. 5, *E* and *F*). In summary, miR-27c-3p plays an important regulatory role in inflammatory responses.

### miR-27c-3p can regulate IRAK4 expression

**Figure 6:**
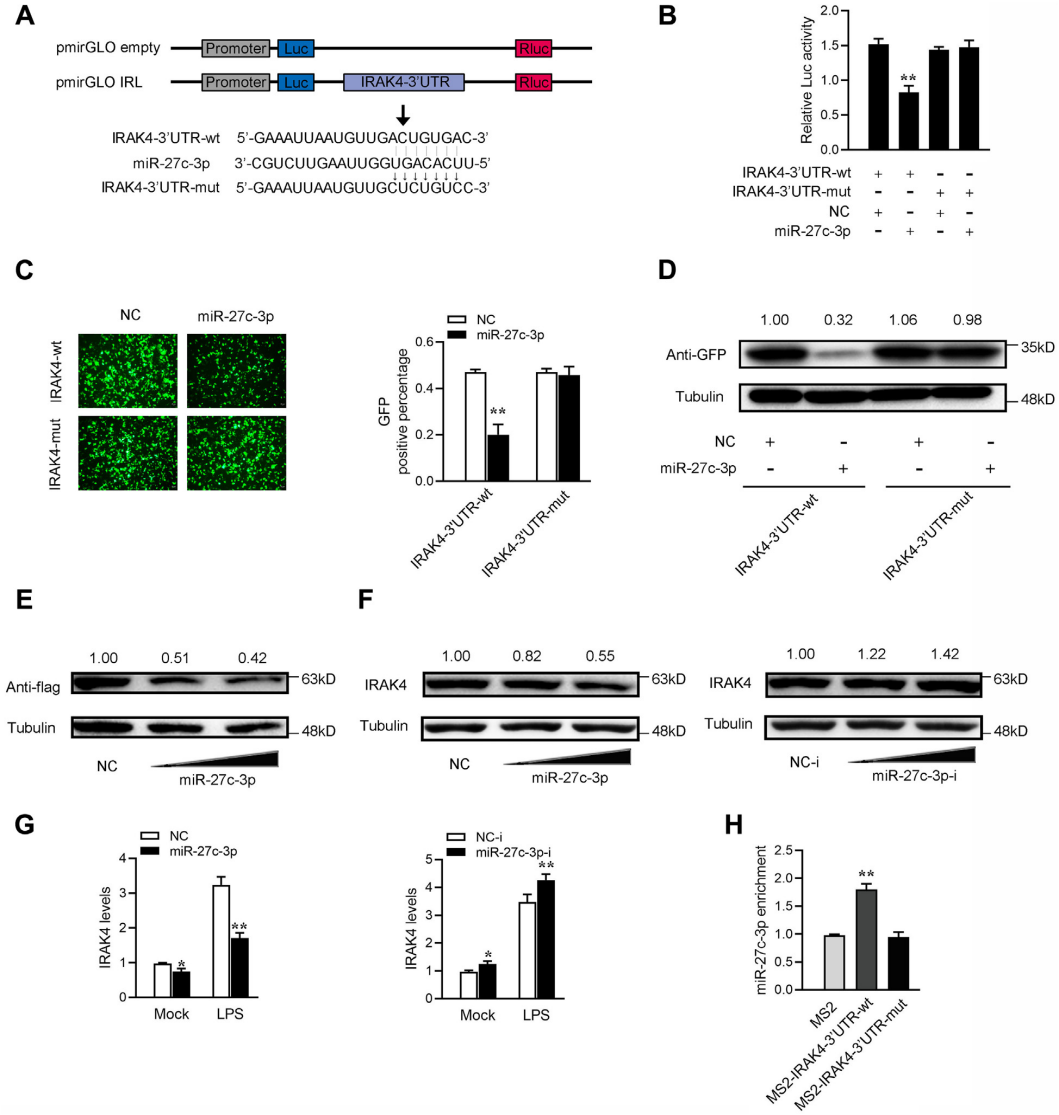
miR-27c-3p can regulate IRAK4 expression. *A*, sequence alignment of miR-27c-3p and its binding sites in the 3’ UTR of IRAK4. miR-27c-3p binding sites in wildtype of IRAK4 3’UTR (IRAK4-3’UTR-wt) and a mutated form of 3’UTR (IRAK4-3’UTR-mut) were shown. *B*, miR-27c-3p target the 3’UTR of IRAK4. EPC cells were transfected with control mimics (NC) or miR-27c-3p mimics (miR-27c-3p), along with IRAK4-3’UTR wt or IRAK4-3’UTR-mut for 48 h, then the luciferase activity was determined. The luciferase activity was measured and normalized to Renilla luciferase activity. *C* and *D*, miR-27c-3p could downregulate GFP expression. EPC cells were cotransfected with the wild type of mVenus-IRAK4-3’UTR or the mutated type of mVenus-IRAK4-3’UTR, together with NC or miR-27c-3p. At 48 h post transfection, the fluorescence intensity (*C*) and the GFP expression levels (*D*) were evaluated by enzyme-labeled instrument and Western blotting, respectively. The scale bar represents 20 μm; original magnification ×10. *E*, miR-27c-3p regulates IRAK4 expression. EPC cells were cotransfected with the IRAK4 expression plasmid, along with miR-27c-3p or NC. After 48 h, IRAK4 expression was determined by Western blotting. *F*, miR-27c-3p suppresses the protein expression of endogenous IRAK4. MICs were cotransfected with miR-27c-3p or NC and miR-27c-3p inhibitors (miR-27c-3p-i) or control inhibitors (NC-i). At 48 h post transfection, the expression of IRAK4 was determined by Western blotting. *G*, miR-27c-3p suppresses the mRNA expression of IRAK4. MICs were cotransfected with miR-27c-3p or NC and miR-27c-3p-i or NC-i. At 48 h post transfection, cells were treated with LPS for 6 h. The expression of IRAK4 was determined by qPCR. *H*, the qPCR results of the MS2-RIP method used to identify the binding between IRAK4-3’UTR and miR-27c-3p in MICs. miRNA real-time qPCR was performed after the RNA immunoprecipitation process. All data represented the mean ± SD from three independent triplicated experiments. ∗*p* < 0.05; ∗∗*p* < 0.01. EPC, epithelioma papulosum cyprinid.

We analyzed the sequence of IRAK4 3’UTR to investigate whether IRAK4 3’UTR can directly interact with miR-27c-3p and find a binding site for miR-27c-3p. Then, we constructed the IRAK4 3’UTR luciferase plasmid and the mutant plasmid with the mutated miR-27c-3p-binding site (Fig. 6*A*). The luciferase assay showed that miR-27c-3p mimics can significantly inhibit the wildtype IRAK4 3’UTR luciferase plasmid activity but they have no effect on the mutated form (Fig. 6*B*). In addition, we inserted the wildtype or the mutant type fragment of IRAK4 3’UTR into the mVenus-C1 vector and cotransfected with miR-27c-3p to confirm whether miR-27c-3p can inhibit the levels of GFP. As shown in Figure 6*C*, miR-27c-3p can significantly inhibit the levels of GFP but cannot inhibit the level of the mutant type of IRAK4-3’UTR-GFP. The Western blotting assay has been conducted to examine the expression level of GFP protein for extending the findings (Fig. 6*D*). These results indicate that the IRAK4 3’UTR may interact with miR-27c-3p through the predicted miR-27c-3p-binding site. We cotransfected miR-27c-3p mimics and IRAK4 expression plasmid with 3’UTR into EPC cells to test whether miR-27c-3p participates in the regulation of IRAK4 expression. The results from Western blotting showed that transfection of miR-27c-3p mimics suppressed the expression levels of IRAK4 (Fig. 6*E*). We transfected miR-27c-3p mimics and inhibitors into MICs. The results from Western blot assays displayed that transfection of miR-27c-3p mimics suppressed the expression levels of IRAK4, whereas miR-27c-3p inhibitors markedly enhanced the expression levels of IRAK4 (Fig. 6*F*). We find through qPCR detection that miR-27c-3p mimics suppressed the expression levels of IRAK4 under LPS treatment, whereas miR-27c-3p inhibitors markedly enhanced the expression levels of IRAK4 under LPS treatment (Fig. 6*G*). We constructed plasmids that recognize IRAK4 3’UTR by MS2 protein. We inserted the MS2-12X fragment into the pcDNA3.1, pcDNA3.1-IRAK4 3’UTR, and the mutated type of IRAK4 3’UTR plasmids (pcDNA3.1-IRAK4-3’UTR-mut). If miRNAs bind to IRAK4, then miR-27c-3p can be pulled down by the GFP-MS2-12X complex. Analysis of qPCR results from the RIP assay showed that the miR-27c-3p enrichment of IRAK4 is significantly higher than that of mutant IRAK4 3’UTR and empty plasmid (Fig. 6*H*). In summary, these results indicated that miR-27c-3p can directly bind to IRAK4 3’UTR and regulate the expression of IRAK4.

### IRL acts as a sponge for miR-27c-3p to enhance IRAK4 expression

**Figure 7:**
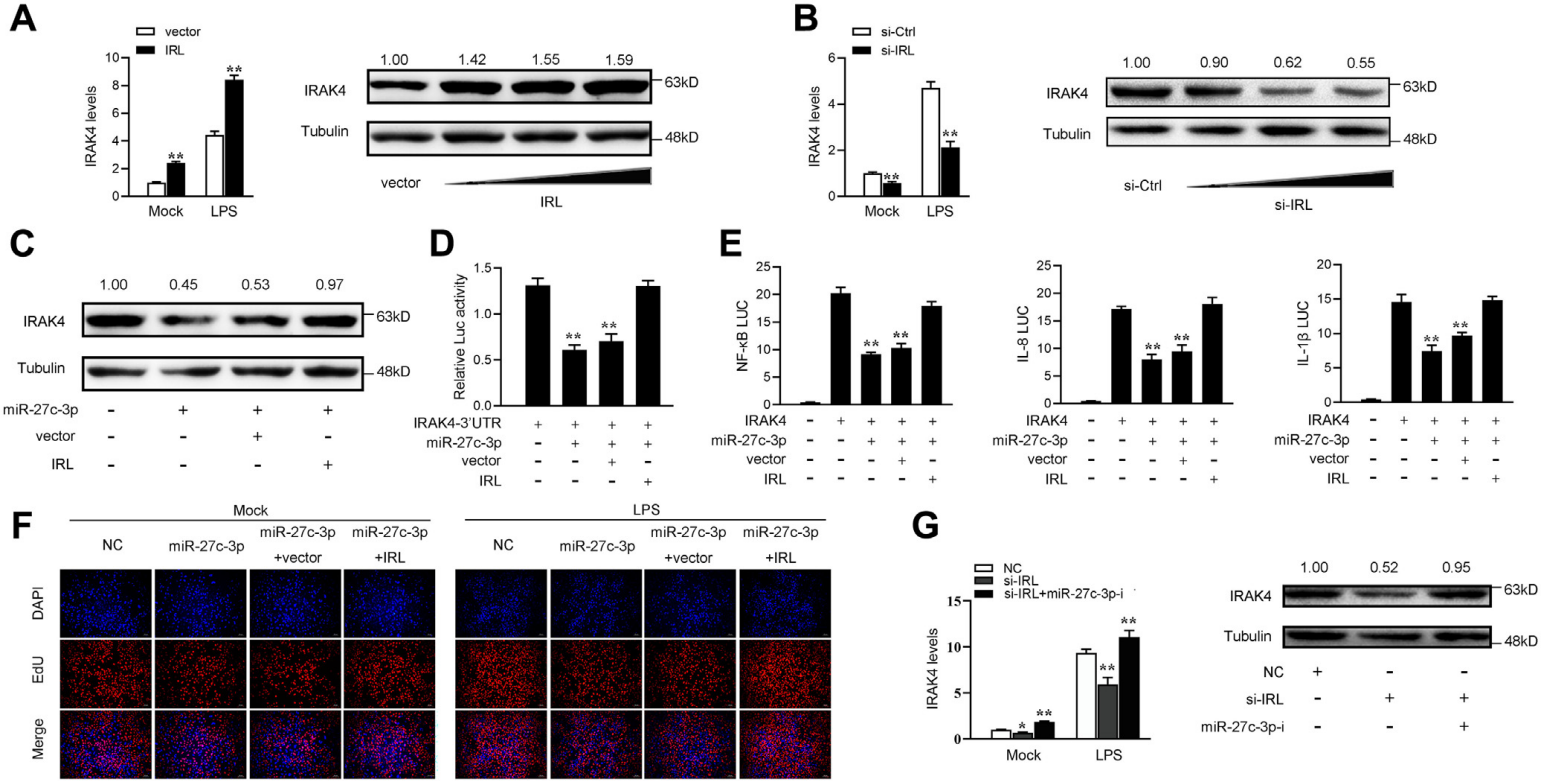
**IRL acts as a sponge for miR-27c-3p to enhance IRAK4 expression.***A* and *B*, relative mRNA and protein levels of IRAK4 in MICs after cotransfection with si-NC or si-IRL and pcDNA3.1 vector or IRL expression plasmid by Western blot assays (*A*) and qPCR (*B*). *C*, Western blot assays were detected in MICs after cotransfection with IRAK4 overexpression plasmid, NC, miR-27c-3p, or IRL. *D*, the relative luciferase activities were detected in epithelioma papulosum cyprinid cells after cotransfection with IRAK4 3’UTR luciferase reporter vector, NC, miR-27c-3p, or IRL. *E*, the relative luciferase activities were detected in MICs after cotransfection with IRAK4 expression plasmid, phRL-TK Renilla luciferase plasmid, luciferase reporters, NC, miR-27c-3p, or IRL. *F*, cell proliferation was assessed by EdU assays in MICs after cotransfection with NC, miR-27c-3p, or IRL. *G*, relative mRNA and protein levels of IRAK4 in MICs after cotransfection with NC, si-IRL and si-IRL, or miR-27c-3p-i by qPCR (*left panel*) and Western blot assays (*right panel*). All data represented the mean ± SD from three independent triplicated experiments. ∗*p* < 0.05; ∗∗*p* < 0.01. IRL, IRAK4-related lncRNA; MIC, *M. miiuy* intestine cell.

Given that IRL interacts with miR-27c-3p and miR-27c-3p targets and regulates IRAK4. Thus, we tested whether IRL can regulate IRAK4. The results from the Western blot and qPCR suggested that IRAK4 protein expression is significantly increased when IRL was overexpressed in MICs, (Fig. 7*A*). Meanwhile, the IRAK4 expression is significantly decreased through knockdown of IRL (Fig. 7*B*). Then, the MICs were cotransfected with miR-27c-3p and IRL, and the Western blotting assay showed that IRL can reverse the inhibitory effect of miR-27c-3p on IRAK4 protein expression (Fig. 7*C*). These results indicated that miR-27c-3p can inhibit IRAK4-3’UTR luciferase activity, whereas IRL can reverse the inhibitory effect of miR-27c-3p on IRAK4 (Fig. 7*D*). The IRAK4 plasmid with full-length 3’UTR, miR-27c-3p, IRL plasmid, and various reporter gene plasmids were cotransfected into EPC cells. The results showed that IRL can reverse the negative effect of miR-27c-3p on the luciferase activities of NF-κB, IL-8, and IL-1β report genes (Fig. 7*E*). Moreover, we attempted to explore the effect of the IRL/miR-27c-3p regulatory loop on cell proliferation. The results indicated that overexpression of IRL can counteract the negative effect of miR-27c-3p on cell proliferation upon LPS stimulation (Fig. 7*F*). We cotransfected miR-27c-3p-i and si-IRL into MICs to further prove the result that IRL regulates IRAK4 by modulating miR-27c-3p. And the results showed that miR-27c-3p-i can reverse the negative effect of si-IRL on IRAK4 (Fig. 7*G*). Collectively, these data demonstrated that IRL serves as a ceRNA for miR-27c-3p to regulate IRAK4 expression.

### The ceRNA network of regulating IRAK4 is widely found in teleost fish

**Figure 8:**
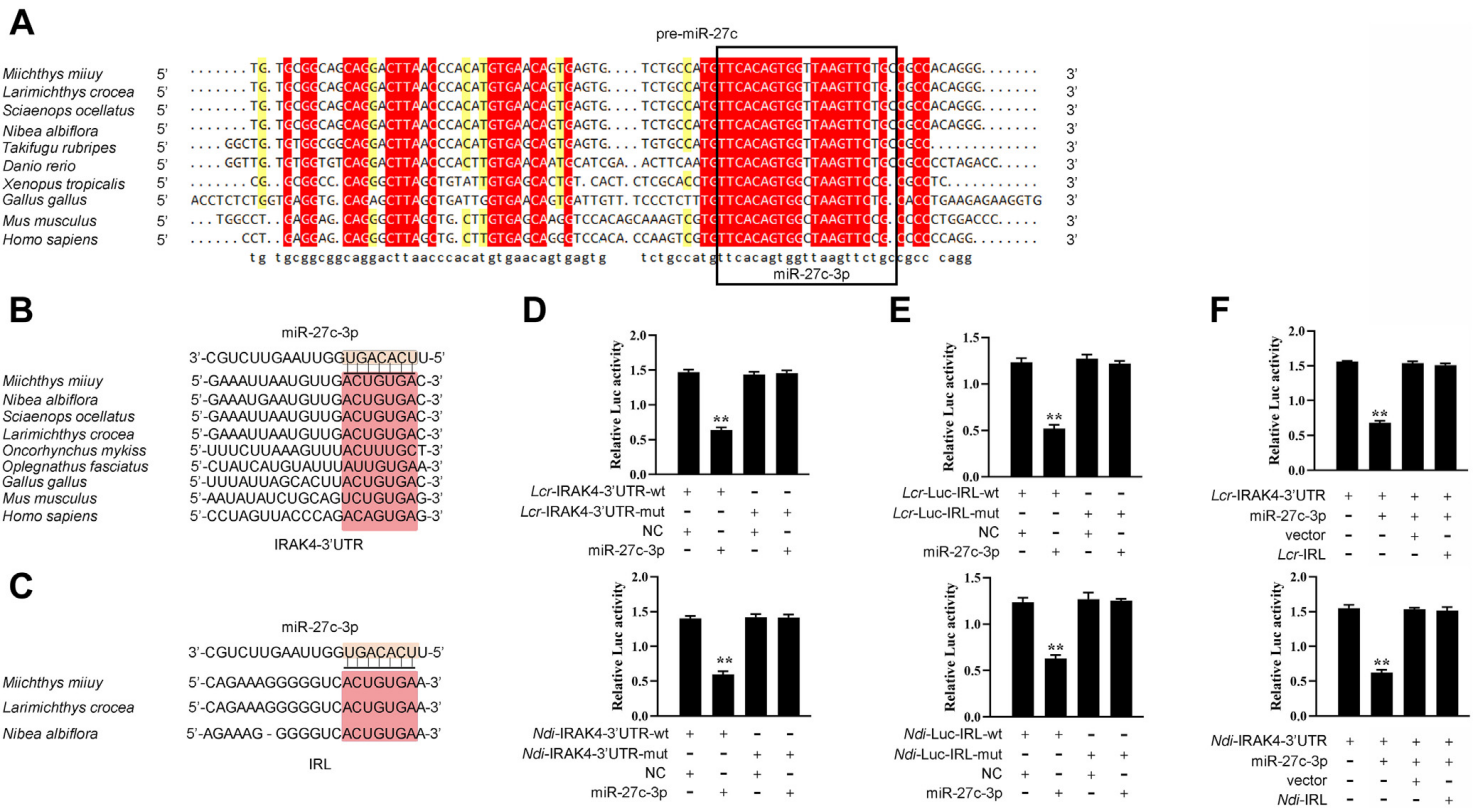
**The competing endogenous RNA network of regulating IRAK4 is widely found in teleost fish.***A*, sequence alignment of pre-miR-217 from various vertebrate species. Mature miR-217 sequences are shown in boxes. *B*, putative miR-27c-3p-binding site of IRAK4 3’UTR among different vertebrate species. *C*, putative miR-27c-3p-binding site of IRL among different vertebrate species. *D*, the relative luciferase activities were detected in EPC cells after cotransfection with *Lcr*-IRAK4-wt or *Lcr*-IRAK4-mut and miR-27c-3p or NC (*upper panel*) and in cells after cotransfection with *Ndi*-IRAK4-wt or *Ndi*-IRAK4-mut and miR-27c-3p or NC (*lower panel*). *E*, the relative luciferase activities were detected in EPC cells after cotransfection with *Lcr*-IRL-wt or *Lcr*-IRL-mut and miR-27c-3p or NC (*upper panel*) and cells after cotransfection with *Ndi*-IRL-wt or *Ndi*-IRL-mut and miR-27c-3p or NC (*lower panel*). *F*, the relative luciferase activities were detected in EPC cells after cotransfection with *Lcr*-IRAK4- 3’UTR luciferase reporter vector, NC, miR-27c-3p, or *Lcr*-IRL (*upper panel*) and EPC cells after cotransfection with *Ndi*-IRAK4-3’UTR luciferase reporter vector, NC, miR-27c-3p, or *Ndi*-IRL (*lower panel*). All data represented the mean ± SD from three independent triplicated experiments. ∗*p* < 0.05; ∗∗*p* < 0.01. EPC, epithelioma papulosum cyprinid; IRL, IRAK4-related lncRNA.

We first examined the sequence alignment of pre-miR-27c from different vertebrate species to address the generality of our findings. Of interest, mature miR-27c-3p as shown in Figure 8*A* displays high conservation from mammals to fish. The miR-27c-3p-binding site in IRAK4 3’UTR also displays high conservation from mammals to fish (Fig. 8*B*). Luciferase report plasmids were generated by cloning the IRL of *L. crocea* and *N. diacanthus* into pmirGLO vector, within the mutant devoid of miR-27c-3p-binding site as a control (Fig. 8*C*), to obtain the direct evidence that miR-27c-3p could target IRL across fish species. It is striking that miR-27c-3p mimics are sufficient to decrease luciferase activities when respectively cotransfected with the wild types of *L. crocea* and *N. diacanthus* IRAK4-3’UTR, whereas they exhibit no effect on the luciferase activity of cells transfected with their mutant types (Fig. 8*D*). These results indicate that miR-27c-3p can target the IRAK4 gene in other fish species. This ability verifies that miR-27c-3p is highly conserved among different species and its function is also conserved to some extent.

**Figure 9:**
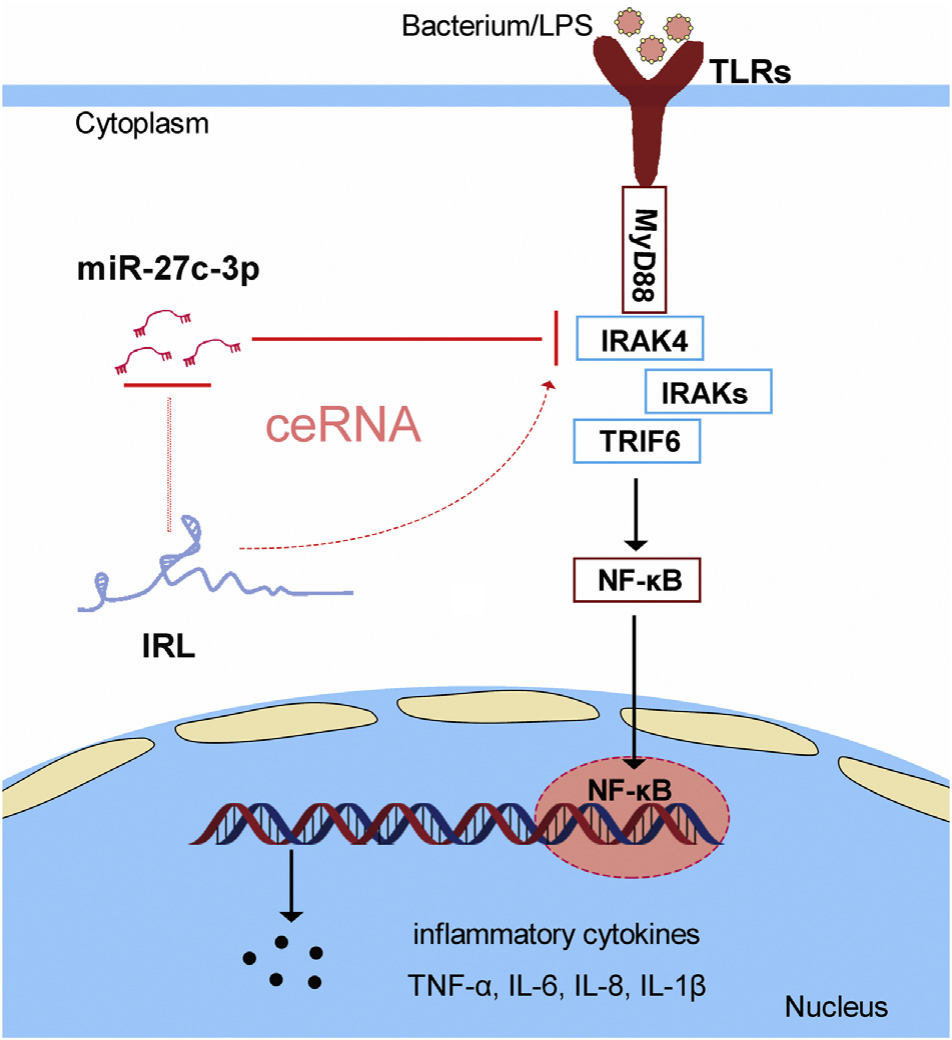
**The mechanism graph of the regulatory network and function of IRAK4.** Fish IRAK4 could induce the innate antibacterial responses through recruiting NF-κB to trigger inflammatory cytokines activation upon bacterial/LPS treatment. miR-27c-3p targets IRAK4 and represses IRAK4-mediated antibacterial responses, thereby helping bacteria resist the host’s antibacterial reaction. IRAK4-related lncRNA acts as a molecular sponge regulating miR-27c-3p to enhance IRAK4 expression, thereby maintaining the stability of antibacterial responses and ensuring appropriate inflammatory responses. The competing endogenous RNA regulatory networks may exist wildly in vertebrate species. LPS, lipopolysaccharide.

We also verified the findings that the interaction between lncRNA IRL and miR-27c-3p also exists in other fish species. To this end, we first examined the sequence alignment of IRL among different species. It is striking that the sequence of IRL is highly conserved among different fish species. In particular, IRL in these different species presents as highly conserved in the binding sites of miR-27c-3p (Fig. 8*C*). Then, we produced luciferase constructs of *L. crocea* and *N. diacanthus* IRL and their mutated forms with mutated miR-27c-3p-binding sites to examine whether IRL in other fish species could also interact with miR-27c-3p. Luciferase assays revealed that miR-27c-3p can suppress the luciferase activity of the wildtype of IRL luciferase plasmid in both fish species but it has no effect on the mutated forms (Fig. 8*E*). Furthermore, to test whether *L. crocea* and *N. diacanthus* IRL can affect miR-27c-3p activity, we conducted luciferase assays and found that both *L. crocea* and *N. diacanthus* IRL could counteract the inhibitory effect of miR-27c-3p on the miR-27c-3p sensor (Fig. 8*F*). These results indicate that IRL may act as endogenous sponge RNA to interact with miR-27c-3p among different fish species (Fig. 9). These data suggest that IRL contains relatively conserved elements among different fish species and this condition is very important for preserving their functions.

## Discussion

Bacterial diseases are one of the most serious threats to the aquaculture industry. In the past decades, various Gram-negative bacteria have been identified as pathogens that result in high mortality in aquaculture species, especially *V. anguillarum*. *V. anguillarum*, as a conditional pathogen that can infect fish and shellfish, is harmful to aquaculture systems and is the primary cause of *Vibrio* infections in various aquatic animals worldwide. In addition, *V. anguillarum* infects aquatic animals by damaging the skin, and has the characteristics of the rapid disease and high mortality, and thus brings serious harm to the aquaculture industry in our country and even the world's aquaculture industry (39). In teleost fish, the innate immune response plays a fundamental and central defense during bacterial infection. Similar to mammals, teleost fish possess conserved immune-relevant genes and a series of signaling events in response to invading bacteria (39). Herein, we discovered an interaction network that positively regulates teleost IRAK4-mediated antibacterial signaling pathways. miR-27c-3p can reduce the expression of IRAK4 and suppress IRAK4-mediated inflammatory responses, and this process will help the bacteria to infect the host cell. Furthermore, we showed that lncRNA IRL acts as an endogenous sponge RNA to interact with miR-27c-3p and enhances the inflammatory responses. Of importance, we find that *V. anguillarum* infection or LPS stimulation could markedly upregulate IRL expression in miiuy croaker. In short, IRL can counteract an increasing effect of miR-27c-3p on LPS stimulation, thus maintaining the stability of antibacterial immune and ensuring appropriate inflammatory responses are guaranteed. The data indicated the critical roles of lncRNAs in modulating inflammatory responses caused by bacterial infection and showed that ceRNA regulation mechanism exists in teleost fish.

At present, the most well-documented PRR is TLRs, and this receptor is effective in detecting various pathogenic characteristic molecules including lipopeptide, peptidoglycan, LPS, flagellin, and nucleic acid (40). After the molecular structures present in pathogens are identified, most TLRs (except TLR3) can activate the NF-κB signaling pathway by activating MyD88 and induce the release of inflammatory cytokines and result in the innate antibacterial response. IRAK4 has been identified as a critical central molecule in the MyD88-mediated NF-κB signaling pathway, and the regulatory mechanism of IRAK4-mediated signal transduction has been extensively investigated in the past few decades (41). In recent years, an increasing number of studies have shown that ncRNAs can regulate the immune response by targeting IRAK4. Zhou *et al.* (42) reported that miR-302b regulates the inflammatory response by feeding back to the TLR/IRAK4 circuit and enhances the defense of the host against Gram-negative bacterium *Pseudomonas aeruginosa* in mice. In addition, Xu *et al.* (43) also reported that miR-93 inhibits inflammatory cytokine production in LPS-stimulated murine macrophages by targeting IRAK4 in mice. Several small ncRNAs that can regulate the immune response mediated by IRAK4 have been recently discovered in lower vertebrates. For example, studies have shown that miR-203 and miR-21 inhibit the inflammatory response upon Gram-negative bacteria or LPS by targeting IRAK4 in fish (16, 44). The present study demonstrated that two novel ncRNAs, namely, miR-27c-3p and IRL, play critical regulatory roles in host inflammatory responses upon LPS stimulation in teleost. Further investigations showed that miR-27c-3p plays a negative role, while IRL exhibits a positive regulatory role in the IRAK4-mediated singling pathway. However, we found that si-IRL did not achieve the desired inhibitory effect on some proinflammatory cytokines. We believe that there may be two reasons for this; on the one hand, it is possible that IRL itself is also able to promote or repress the expression of these proinflammatory cytokines by driving the expression of other molecules, thus influencing the regulation of these cytokines by this axis, which is IRL-miR-27c-3p-IRAK4. On the other hand, we believe that this result is caused by the inhibitory efficiency of siRNA.

During the last decades, the research on the regulatory network mechanism of miRNAs is increasingly becoming more and more complete and clear in mammals, while the research on miRNAs in lower vertebrates (especially in the teleost fish) is also constantly deepening. Clearly, complex miRNA regulation networks exist in teleost fish in regulating innate immunity. For example, the inducible miR-203 and miR-21 have been reported to represses the inflammatory responses to Gram-negative bacteria by targeting IRAK4 (16, 44). miR-214 and miR-3570 have been reported to modulate NF-κB-mediated inflammatory response via targeting MyD88 gene in miiuy croaker (32, 45). Furthermore, recent studies showed that miRNA can interact with lncRNA to form a regulatory network for regulating the immune response. In miiuy croaker, miR-122 can regulate MAVS expression and suppress MAVS-mediated antiviral responses, while lncRNA MARL can function as a ceRNA for miR-122 to maintain the stability of antiviral responses; this finding indicates the presence of lncRNA-miRNA interaction in lower vertebrates (34). In this study, miR-27c-3p is proven to be a novel miRNA targeting IRAK4 in miiuy croaker. miR-27c-3p negatively regulates IRAK4 expression and suppresses IRAK4-mediated antibacterial responses. The negative regulatory mechanism may be a strategy of Gram-negative bacteria for their survival by resisting the host antibacterial immune response.

Accumulating pieces of evidence suggested that some lncRNAs can play as a ceRNA to regulate the expression of coding genes in mammals by competitively sponging the miRNAs. This ceRNA mechanism was first discovered in plants and has since been widely confirmed in mammals, and most lncRNAs with this function are mainly distributed in the cytoplasm (46, 47). For example, cytoplasm lncRNA LINC00963 confers oncogenic function in the progression of glioma and that of a ceRNA net of LINC00963-miR-506-BCAT1 (48). Moreover, the cytoplasm lncRNA LINC01133 has been reported to act as a ceRNA for inhibiting gastric cancer progression by sponging miR-106a-3p to regulate APC expression and the Wnt/β-catenin pathway (49). In addition, lncRNA MIAT is significantly increased in Ang II-induced cardiac hypertrophy and contributes to the pathological development by suppressing miR-150 expression in cardiomyocytes; moreover, miR-150 is a downstream effector of MIAT in the development of cardiac hypertrophy (50). Although a large number of lncRNAs have been found in lower vertebrates, the biological function and significance of lncRNAs in lower vertebrates are still poorly understood unlike those in mammals. Our study showed that lncRNA IRL can act as a ceRNA to regulate IRAK4-mediated antibacterial pathway through competitively adsorbing miR-27c-3p in teleost fish. This study is the first one to report that lncRNA can regulate antibacterial pathways mediated by TLRs through the ceRNA mechanism in lower vertebrates, which will benefit for understanding vertebrate immunology and the evolution of immune systems among vertebrates.

In summary, we find that miR-27c-3p plays a negative role, whereas IRL exhibits a positive regulatory role in the regulation of inflammatory responses. Furthermore, we identified that IRL acts as a regulator of inflammatory responses via acting as a ceRNA for miR-27c-3p to relieve its repressive effects on IRAK4 expression; thus, immune homeostasis and immune balance are maintained. Our findings suggest the critical roles of lncRNAs in operating fish inflammatory response processes, which will benefit for understanding the vertebrate immunology and the evolution of immune systems among vertebrates.

## Data availability

All data are contained within the article and the supporting information.

## Conflict of interest

The authors declare that they have no conflict of interest with the contents of this article.
