# Supplementary material for: Long noncoding RNA IRL regulates NF-κB-mediated immune responses through suppression of miR-27c-3p-dependent IRAK4 downregulation in teleost fish
Source: J Biol Chem. 2021 Jan 16;296:100304. doi: 10.1016/j.jbc.2021.100304 (PMC7949060; doi:10.1016/j.jbc.2021.100304)
Supplement: Supplementary file 1 — Figures S1 & S2 and Tables S1 & S2 [file mmc1.pdf]

Long noncoding RNA IRL regulates NF-κB-mediated immune responses through suppression of miR-27c-3p-dependent IRAK4 down-regulation in teleost fish

Weiwei Zheng[1,2], Qing Chu[1,2], Tianjun Xu[1,2,3,4,*]

1. *Laboratory of Fish Molecular Immunology, College of Fisheries and Life Science, Shanghai Ocean University, Shanghai, China*
2. *Laboratory of Marine Biology and Biotechnology, Qingdao National Laboratory for Marine Science and Technology, Qingdao, China*
3. *Key Laboratory of Exploration and Utilization of Aquatic Genetic Resources (Shanghai Ocean University), Ministry of Education, China*
4. *National Pathogen Collection Center for Aquatic Animals, Shanghai Ocean University, Shanghai, China*

*Corresponding author. Dr. Tianjun Xu

E-mail: tianjunxu@163.com

Running title: lncRNA regulates immune responses in fish

Supplementary Materials

Supplemental Table 1. PCR primer information in this study.

Primer	Sequence (5'-3')
**Real-time PCR**	
IRAK4-RT-F	ATCGGCTAAGCGGACATCA
IRAK4-RT-R	TACCTCGCCTCCATCAAGA
IRL-RT-F	ACCGCAACAAGGGATT
IRL-RT-R	TCCGCACAGGGAATAA
TNF-α-RT-F	GTTTGCTTGGTACTGGAATGG
TNF-α-RT-R	TGTGGGATGATGATCTGGTTG
IL-1β-RT-F	CATAAGGATGGGGACAACGAG
IL-1β-RT-R	TAGGGGACGGACACAAGGGTA
IL-6-RT-F	GCGGTAAAGGCATGGATAT
IL-6-RT-R	GTTGTAGTTGGAAGGGCAG
IL-8-RT-F	AGCAGCAGAGTCTTCGT
IL-8-RT-R	TCTTCGCAGTGGGAGTT
GAPDH-RT-F	ACCTTCACTCCTCCATCTT
GAPDH-RT-R	AGGTCACAGACACGGTTG
miR-27c-3p-RT-F	GCAGTTCACAGTGGTTAAGTTC
miR-27c-3p-RT-R	TCCAGTTTTTTTTTTTTTTTGCAGA
5.8S-RT-F	AACTCTTAGCGGTGGATCA
5.8S-RT-R	GTTTTTTTTTTTTTTTGCCGAGTG
**Vector construction**	
IRAK4-pcDNA3.1-F	CGCGGATCCAATAATTCAGTAACTTCCGC
IRAK4-pcDNA3.1-R	AACTCTGGATTTGCTGCAG
IRAK4-3'UTR-F	CGAGCTCCGTAGCATCCCAGAG
IRAK4-3'UTR-R	TGCTCTAGACAAGATATTAAGCCATC
IRAK4-3'UTR-mut-F	GTTGCTATAACCAAATGTATTCTTTTGCAACAATCTTT
IRAK4-3'UTR-mut-R	CATTTGGTTATAGCAACATTAATTTCTCTAAATCTGCTGAA
IRAK4-3'UTR-mVenus-F	CCCAAGCTTCGTAGCATCCCAGAG
IRAK4-3'UTR-mVenus-R	CGCGGATCCCAAGATATTAAGCCATC
IRAK4-3'UTR-mVenus-mut-F	GTTGCTATAACCAAATGTATTCTTTTGCAACAATCTTT
IRAK4-3'UTR-mVenus-mut-R	CATTTGGTTATAGCAACATTAATTTCTCTAAATCTGCTGAA
pcDNA3.1-MS2-IRAK4-3'UTR-F	CCCAAGCTTCGTAGCATCCCAGAG
pcDNA3.1-MS2-IRAK4-3'UTR-R	CGCGGATCCCAAGATATTAAGCCATC
IRL-pcDNA3.1-F	ACTATAGGGAGACCCAAGCTTGTCATCACTGTCACATCGTTAATATCTAC
IRL-pcDNA3.1-R	GCGGCCGTTACTAGTGGATCCTCCGCACAGGGAATAAGGAG
IRL-pmirGLO-F	TGTTTAAACGAGCTCGCTAGCGTCATCACTGTCACATCGTTAATATCTAC
IRL-pmirGLO-R	CAGGTCGACTCTAGACTCGAGTCCGCACAGGGAATAAGGAG
IRL-pmirGLO-mut-F	GGGTCCTATAACAAGAGTCCGGCGCGCTTTTT
IRL-pmirGLO-mut-R	CTCTTGTTATAGGACCCCCTTTCTGAAATCCCT

IRL-mVenus-F	TCAGATCTCGAGCTCAAGCTTGTCATCACTGTCACATCGTTAATATCTAC
IRL-mVenus-R	CGGGCCCGCGGTACCGTCGACTCCGCACAGGGAATAAGGAG
IRL-mVenus-mut-F	GGGTCCTATAACAAGAGTCCGGCGCGCTTTTTC
IRL-mVenus-mut-R	CTCTTGTTATAGGACCCCCTTTCTGAAATCCCT
pcDNA3.1-MS2-IRL-F	ACTATAGGGAGACCCAAGCTTGTCATCACTGTCACATCGTTAATATCTAC
pcDNA3.1-MS2-IRL-R	GCGGCCGTTACTAGTGGATCCTCCGCACAGGGAATAAGGAG
pcDNA3.1-MS2-IRL-mut-F	GGGTCCTATAACAAGAGTCCGGCGCGCTTTTTC
pcDNA3.1-MS2-IRL-mut-R	CTCTTGTTATAGGACCCCCTTTCTGAAATCCCT
IRL-T7-F	TAATACGACTCACTATAGGGGTCATCACTGTCACATCGT
IRL-T7-R	TCCGCACAGGGAATAA
Lcr-IRAK4-3'UTR-F	CGAGCTCCGTAGCATCCCAGAG
Lcr-IRAK4-3'UTR-R	TGCTCTAGACAAGATATTAAGCCATC
Lcr-IRAK4-3'UTR-mut-F	GTTGCTATAACCAAATGTATTCTTTTGCAACAATCTTT
Lcr-IRAK4-3'UTR-mut-R	CATTTGGTTATAGCAACATTAATTTCTCTAAATCTGCTGAA
Lcr-IRL-pcDNA3.1-F	ACTATAGGGAGACCCAAGCTTGTCATCACTGTCACATCGTTAATATCTAC
Lcr-IRL-pcDNA3.1-R	GCGGCCGTTACTAGTGGATCCTCCGCACAGGGAATAAGGAG
Lcr-IRL-pmirGLO-F	TGTTTAAACGAGCTCGCTAGCGTCATCACTGTCACATCGTTAATATCTAC
Lcr-IRL-pmirGLO-R	CAGGTCGACTCTAGACTCGAGTCCGCACAGGGAATAAGGAG
Lcr-IRL-pmirGLO-mut-F	GGGTCCTATAACAAGAGTCCGGCGCGCTTTTTC
Lcr-IRL-pmirGLO-mut-R	CTCTTGTTATAGGACCCCCTTTCTGAAATCCCT
Ndi-IRAK4-3'UTR-F	CGAGCTCCGTAGCATCCCAGAG
Ndi-IRAK4-3'UTR-R	TGCTCTAGACAAGATATTAAGCCATC
Ndi-IRAK4-3'UTR-mut-F	GTTGCTATAACCAAATGTATTCTTTTGCAACAATCTTT
Ndi-IRAK4-3'UTR-mut-R	CATTTGGTTATAGCAACATTAATTTCTCTAAATCTGCTGAA
Ndi-IRL-pcDNA3.1-F	ACTATAGGGAGACCCAAGCTTGTCATCACTGTCACATCGTTAATATCTAC
Ndi-IRL-pcDNA3.1-R	GCGGCCGTTACTAGTGGATCCTCCGCACAGGGAATAAGGAG
Ndi-IRL-pmirGLO-F	TGTTTAAACGAGCTCGCTAGCGTCATCACTGTCACATCGTTAATATCTAC
Ndi-IRL-pmirGLO-R	CAGGTCGACTCTAGACTCGAGTCCGCACAGGGAATAAGGAG
Ndi-IRL-pmirGLO-mut-F	GGGTCCTATAACAAGAGTCCGGCGCGCTTTTTC
Ndi-IRL-pmirGLO-mut-R	CTCTTGTTATAGGACCCCCTTTCTGAAATCCCT
miR-27c-3p-sensor-F	TCGAGAAGTGTCACCAATTCAAGACGAAGTGTCACCAATTCAAGACGGC
miR-27c-3p-sensor-R	GGCCGCCGTCTTGAATTGGTGACACTTCGTCTTGAATTGGTGACACTTC

Supplemental Table 2. The predicted miR-27c-3p targets using bioinformatics

miRNA	Prediction of miRNA Targets
miR-27c-3p	E3 ubiquitin-protein ligase Itchy homolog
	RAC-alpha serine/threonine-protein kinase
	Interleukin-1 receptor-associated kinase 4
	Autophagy protein 5
	Serine-protein kinase ATM
	Bcl2-associated agonist of cell death
	BH3-interacting domain death agonist
	Bone morphogenetic protein 1
	Growth/differentiation factor 2
	Bone morphogenetic protein receptor type-1B
	Laminin subunit gamma-2
	Calreticulin
	C-C chemokine receptor type 5
	Cdc42 homolog
	Cathepsin S
	Dedicator of cytokinesis protein 2
	Phosphoenolpyruvate carboxykinase, cytosolic
	EWS/FLI1-activated transcript 2
	Eukaryotic translation initiation factor 4E
	Cell death abnormality protein 12
	Receptor protein-tyrosine kinase
	Vascular endothelial growth factor receptor 1
	Fibronectin
	Growth arrest-specific protein 6
	Growth hormone receptor
	Guanine nucleotide-binding protein subunit alpha-12
	Guanine nucleotide-binding protein subunit beta-5
	Guanine nucleotide-binding protein G(T) subunit gamma-T1
	GTPase HRas
	Intercellular adhesion molecule 2
	Gamma-interferon-inducible lysosomal thiol reductase

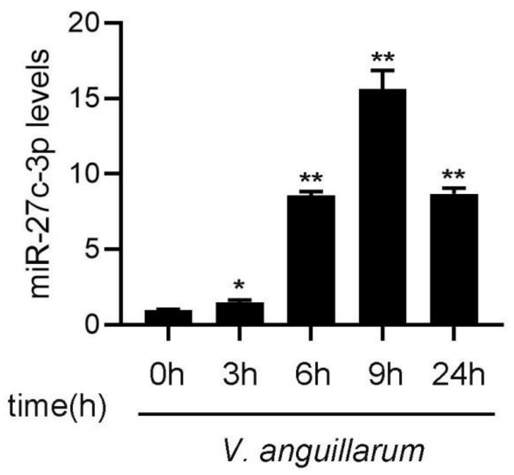

Figure S1. *V. anguillarum* induce the expression of the miR-27c-3p. The expression levels of miR-27c-3p in spleen samples were measured by qPCR after *V. anguillarum* infection. All data represented the mean ± SD from three independent triplicated experiments. *, $p < 0.05$.

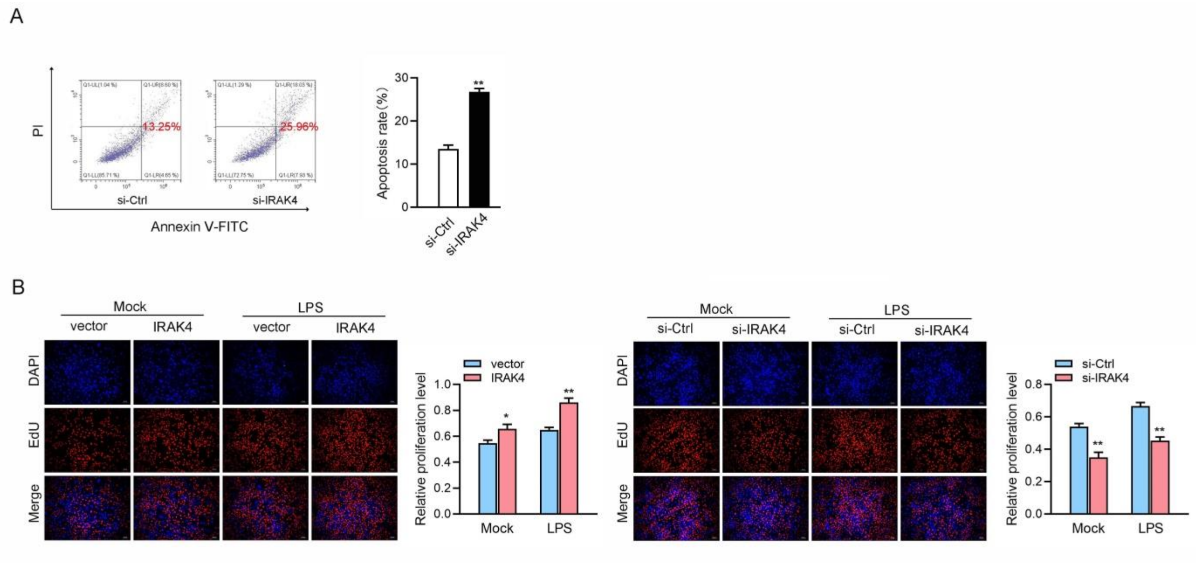

Figure S2. IRAK4 can promote cell proliferation and inhibit cell apoptosis. (A) The effect of IRAK4 knockdown on cell apoptosis was analyzed by flow cytometric cell apoptosis assays. (B) Cell proliferation was assessed by EdU assays in MIC cells after cotransfected with vector or IRAK4 expression plasmid and si-NC or si-IRAK4 after LPS stimulateion. All data represented the mean ± SD from three independent triplicated experiments. *, $p < 0.05$; **, $p < 0.01$.
